# Impacts of stress hyperglycemia ratio on early neurological deterioration and functional outcome after endovascular treatment in patients with acute ischemic stroke

**DOI:** 10.3389/fendo.2023.1094353

**Published:** 2023-01-26

**Authors:** Zheng Dai, Haiming Cao, Feng Wang, Lei Li, Hongquan Guo, Xiaohao Zhang, Haichang Jiang, Juehua Zhu, Yongjun Jiang, Dezhi Liu, Gelin Xu

**Affiliations:** ^1^ Department of Neurology, Jinling Hospital, Nanjing Medical University, Nanjing, China; ^2^ Department of Neurology, Affiliated Wuxi People’s Hospital of Nanjing Medical University, Wuxi, China; ^3^ Department of Neurology, Nanjing First Hospital, Nanjing Medical University, Nanjing, China; ^4^ Department of Neurology, Jinling Hospital, First School of Clinical Medicine, Southern Medical University, Nanjing, China; ^5^ Department of Neurology, First Affiliated Hospital of Soochow University, Suzhou, China; ^6^ Department of Neurology, Second Affiliated Hospital of Guangzhou Medical University, Guangzhou, China; ^7^ Department of Neurology, Shuguang Hospital Affiliated to Shanghai University of Traditional Chinese Medicine, Shanghai, China; ^8^ Department of Neurology, Shenzhen Second People’s Hospital, Shenzhen, Guangdong, China; ^9^ Department of Neurology, First Affiliated Hospital of Shenzhen University, Shenzhen, Guangdong, China

**Keywords:** acute ischemic stroke, early neurological deterioration, endovascular thrombectomy, large artery occlusion, stress hyperglycemia ratio

## Abstract

**Background and Purpose:**

Hyperglycemia has been associated with unfavorable outcome of acute ischemic stroke, but this association has not been verified in patients with endovascular thrombectomy treatment. This study aimed to assess the impact of stress hyperglycemia ratio on early neurological deterioration and favorable outcome after thrombectomy in patients with acute ischemic stroke.

**Methods:**

Stroke patients with endovascular thrombectomy in two comprehensive centers were enrolled. Early neurological deterioration was defined as ≥4 points increase of National Institutes of Health Stroke Scale (NIHSS) at 24 hours after endovascular procedure. Favorable outcome was defined as modified Rankin Scale (mRS) score of 0-2 at 90 days of stroke onset. Multivariate regression analysis was used to identify the predictors for early neurological deterioration and favorable outcome.

**Results:**

Among the 559 enrolled, 74 (13.2%) patients developed early neurological deterioration. The predictors for early neurological deterioration were high stress hyperglycemia ratio at baseline (OR =5.77; 95% CI, 1.878-17.742; *P* =0.002), symptomatic intracranial hemorrhage (OR =4.90; 95% CI, 2.439-9.835; *P <*0.001) and high NIHSS score after 24 hours (OR =1.11; 95% CI, 1.071-1.151; *P <*0.001). The predictors for favorable outcome were stress hyperglycemia ratio (OR =0.196, 95% CI, 0.077-0.502; *P* =0.001), age (OR =0.942, 95% CI, 0.909-0.977; *P* =0.001), NIHSS score 24 hours after onset (OR =0.757, 95% CI =0.693-0.827; P <0.001), groin puncture to recanalization time (OR =0.987, 95% CI, 0.975-0.998; *P* =0.025), poor collateral status before treatment (ASITN/SIR grade 0-3, OR =62.017, 95% CI, 25.920-148.382; *P <*0.001), successful recanalization (mTICI 2b or 3, OR =7.415, 95% CI, 1.942-28.313; *P* =0.001).

**Conclusion:**

High stress hyperglycemia ratio may be related to early neurological deterioration and decreased likelihood of favourable outcomes after endovascular thrombectomy in patients with acute ischemic stroke.

## Introduction

Endovascular thrombectomy has been involving as the first-line treatment for acute ischemic stroke caused by large artery occlusion (1–3). However, mechanical recanalization not always necessarily resulted in favorable outcome even when patients were treated within 6 hours of stroke onset (4, 5). Exploring the possible factors associated with early neurological deterioration (END), a strong predictor for functional outcomes, is of vital importance for continuously improving the efficacy of endovascular thrombectomy in stroke patients (6, 7).

Hyperglycemia was associated with END in patients with acute ischemic stroke (8–10). Hyperglycemia could destruct blood-brain barrier, aggravate ischemic lesion, increase risk of hemorrhage transformation after cerebral infarction, and reduce duration of ischemic penumbra existence (11–14). Glycated hemoglobin (HbA1c) is more stable than blood glucose level in patients with acute ischemic stroke, and stress hyperglycemia ratio, defined as the stress fasting glycemia/HbA1c ratio (SHR), may be more feasible for evaluating the functional outcome. Some studies observed that stroke patients with high SHR had decreased likelihood of favorable functional outcome and increased likelihood of recurrence and intracranial hemorrhage after recanalization treatment (15–17); others missed these phenomena (18). Therefore, the relationship between SHR and END or functional outcome after endovascular recanalization treatment in patients with acute ischemic stroke is far from determined. This study aimed to investigate the effects of SHR on END and functional outcome in patients with acute ischemic stroke and treated with endovascular thrombectomy.

## Methods

### Study population

Stroke patients with endovascular thrombectomy in two comprehensive centers were screened for eligibility during November 1, 2018 and May 31, 2022. Local ethic review board approved the study protocol. Due to its retrospective nature, patient consent was waived.

Patients were treated with endovascular thrombectomy if they: 1) aged 18 years or old; 2) had ischemic stroke caused by large artery occlusion in anterior or posterior circulation; 3) pre-stroke mRS score ≤2; and 4) had arterial sheath being placed in 6 hours of stroke onset or met the DAWN or DEFUSE criteria (19, 20). Patients were not treated with endovascular thrombectomy if they: 1) had a life expectancy <12 months; 2) had severe cardiopulmonary failure; 3) had a platelet count of <55×1000/mm^3^; or 4) had anemia (hemoglobin <100 g/l) or other conditions which may affect HbA1c measurement.

### Recanalization treatment and baseline assessment

All thrombectomy procedures were performed with Solitaire (Covidien, Irvine, CA) and Catalyst6 devices (Stryker, Kalamazoo, MI) alone or in combination. Successful recanalization was defined as grade 2b-3 in modified thrombolysis in cerebral infarction (mTICI). Collateral circulation was evaluated using the American Society of Interventional and Therapeutic Neuroradiology/Society of Interventional Radiology collateral vessel grading system (ASITN/SIR), and categorized into grade 0 or 1, 2, and 3 or 4. The door to groin puncture time (DPT), groin puncture to final recanalization time (PRT), number of retriever passes, intravenous thrombolysis, and rescue treatment (including angioplasty, stenting, intra-artery thrombolysis) were recorded. Possible pre-procedure infarction were quantified using the Alberta Stroke Program Early CT Score (ASPECTS) or ASPECTS for posterior circulation (pc-ASPECTS) on non-contrast CT. CT was performed immediately and 24 hours after the endovascular procedures to detect possible intracranial hemorrhage. An extra CT scan was arranged whenever as the novel symptoms indicated.

### Follow-up assessment

Stroke severity was assessed using the National Institutes of Health Stroke Scale (NIHSS) score. Stress hyperglycemia ratio was defined as the stress fasting glycemia/HbA1c ratio (SHR). END was defined as an increase of 24-hour NIHSS score of ≥4 points after endovascular procedure (21). Favorable outcome was defined as a mRS score of 0-2 at 90 days of stroke onset. Symptomatic intracranial hemorrhage (sICH) was defined and classified according to the European Cooperative Acute Stroke Study (ECASS-III) criteria (22). Malignant brain edema was defined and classified according to the SITS-MOST (Safe Implementation of Thrombolysis in Stroke-Monitoring Study) protocol, and grade 3 was defined as malignant edema (23, 24).

### Statistical analysis

Categorical variables were expressed as frequencies and percentages, and analyzed with χ2 or Fisher’s exact test. Quantitative variables were expressed as medians and interquartile ranges (IQRs), and were analyzed with Mann-Whitney U test. Receiver Operating Characteristic Curve (ROC) was constructed to explore the cutoff value of SHR for predicting favorable outcome. Multivariable logistical regression model was used to assess the potential factors associated with favorable outcome. Parameters with *P <*0.05 in univariate analysis entered in multivariate analysis. The covariates included in the multivariable logistical regression were mTICI score (2b or 3), MCE, sICH, NIHSS 24 hours after procedure, Pre-procedure ASPCET score, PTR, Pre-procedure ASITN/SIR score, homocysteine, retriever passes >3 times, lymphocyte, HbA1c, fasting blood glucose, glycosylated hemoglobin, SHR. Model 1 and Model 2 were diabetic group and non-diabetic group, respectively. Model 3 included fasting blood glucose and glycosylated hemoglobin as confounders, and model 4 excluded fasting blood glucose and glycosylated hemoglobin as confounders. P value of <0.05 was considered as statistically significant. Statistical analyses were performed using SPSS 25.0 (IBM, Armonk, NY).

## Results

**Figure 1:**
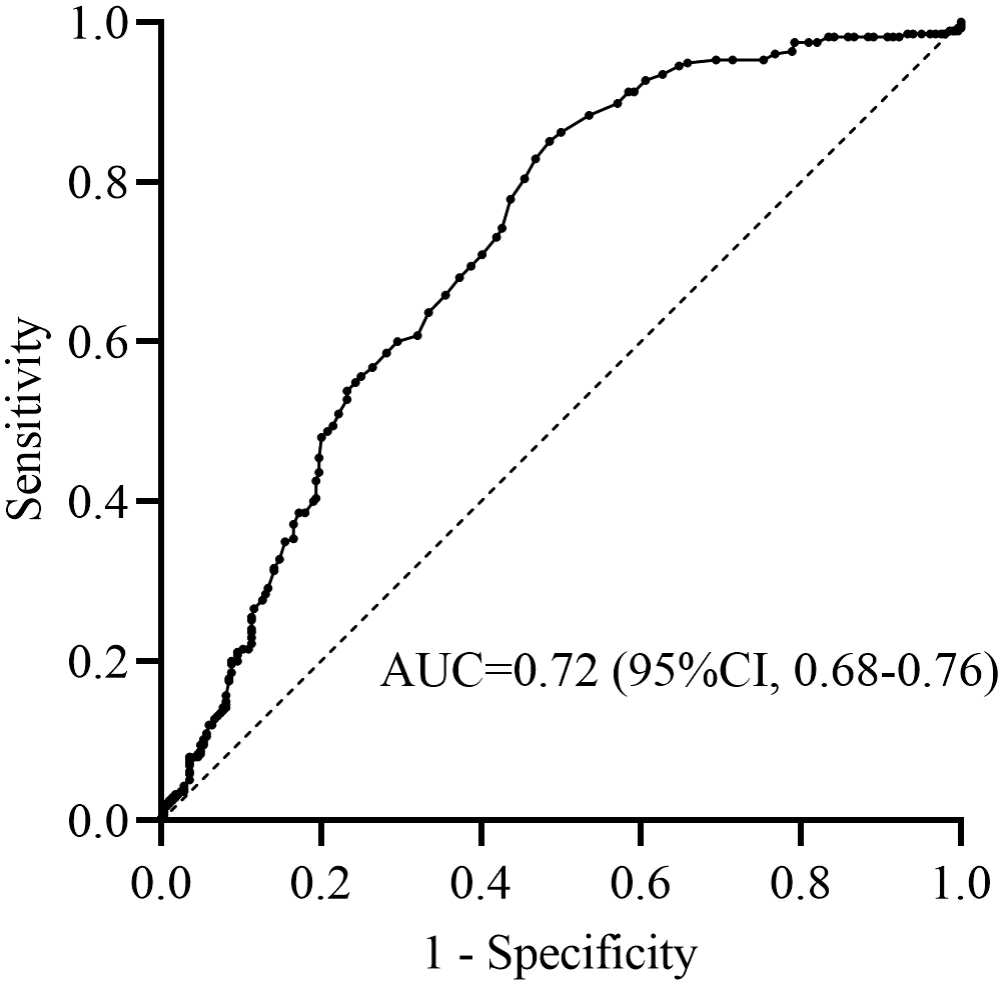
Sensitivity and specificity of SHR in predicting favorable outcome.

A total of 559 stroke patients were enrolled. The median (IQR) age was 70 (63–77) years, and NIHSS score after 24 hours of thrombectomy was 12 (6-19). There were 357 (63.9%) male patients. Among the enrolled, 74 (13.2%) patients developed END. There were 69 (12.3%) patients occurred sICH in 24 hours, and 81 patients (14.5%) died in 90 days. Favorable outcome was obtained in 284 (50.8%) patients. There were 190 (34.0%) patients had high SHR. The ROC curve showed that the optimal cutoff value of SHR for predicting favorable outcome was 1.07, the sensitivity was 84.7%, the specificity was 52.5%, and the Youden index was 0.372 (**Figure 1**).

**Figure 2:**
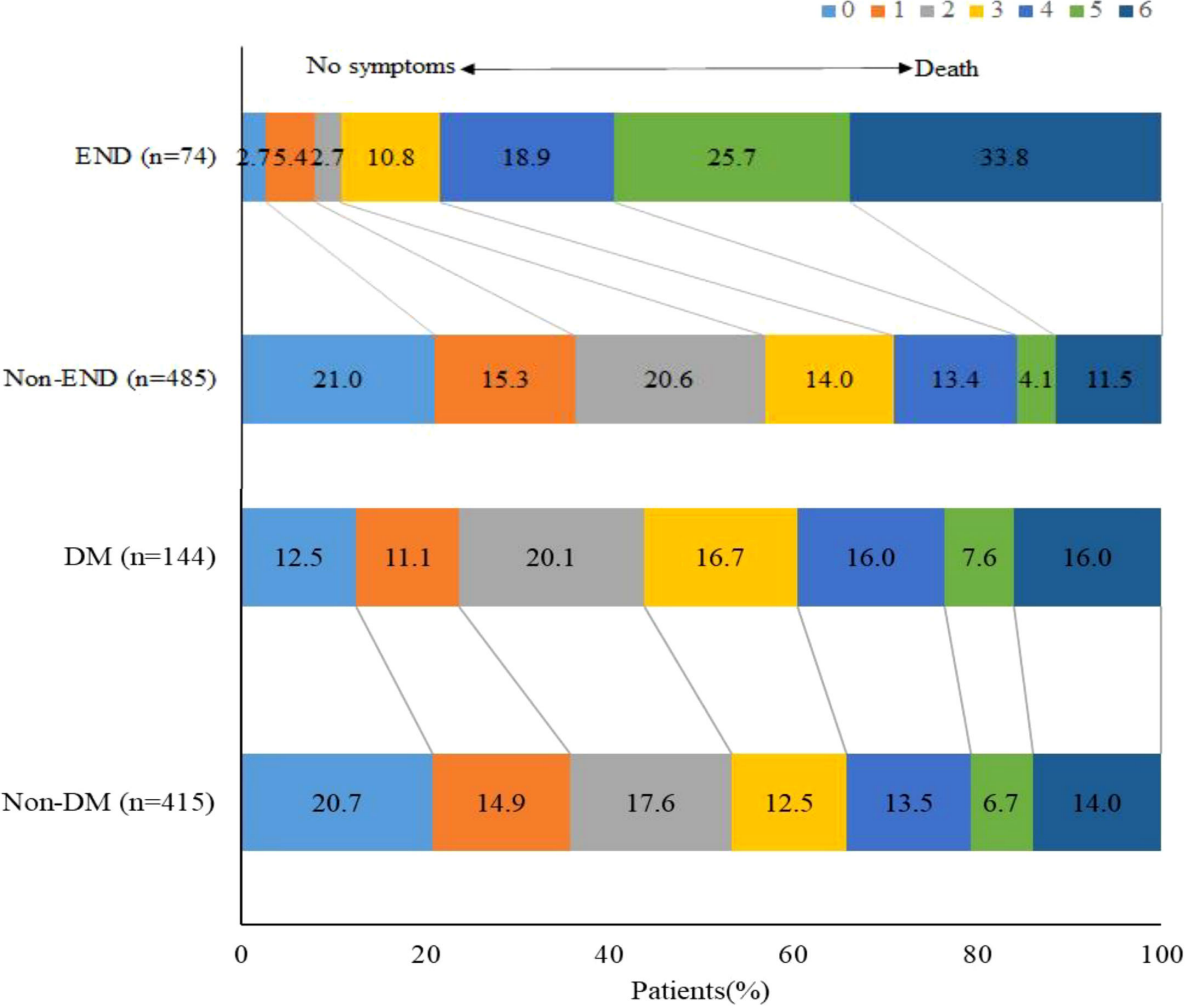
Functional outcomes according to END and DM. Distribution of modified Rankin Scale scores at 90 days.

Compared with patients without END, those with END had higher median NIHSS scores after 24 hours (29 vs. 11, *P <*0.001), higher homocysteine (16.4 vs. 14.6, *P* =0.029), higher fasting glucose levels (8.7 vs. 6.9, *P <*0.001), higher glycated hemoglobin (6.4 vs. 5.9, *P* =0036), higher SHR (1.4 vs. 1.1, *P <*0.001), lower pre-procedure ASPECTS score (8 vs. 9, *P* =0.001), lower pre-procedure ASITN grade (1 vs. 3, *P <*0.001), longer PRT (80 vs. 67, *P* =0.014), higher proportion of multiple retriever passes (16.2% vs. 8.2%, *P* =0.028), lower proportion of successful recanalization (83.8% vs. 91.3%, *P* =0.040), and higher proportion of malignant brain edema (28.4% vs. 15.5%, *P* =0.006). The proportion of sICH was higher (48.6% vs. 6.8%, *P <*0.001) in patient with END than that in patient without. Proportion of favorable outcome was lower (10.8% vs. 56.9%, *P <*0.001), and mortality (33.8% vs. 11.5%, *P <*0.001) was higher in patients with END. Moreover, subgroup analysis showed that proportion of favorable outcome was lower in patients with diabetes mellitus. (**Table 1**, **Figure 2**).

**Table 1:** Baseline characteristics and clinical outcomes according to END.

Variable	END		P value
	with without n=74 n=485		
Age, y, median (IQR)	69 (61-76)	70 (63-77)	0.696
Male, n (%)	49 (66.2)	308 (63.5)	0.651
NIHSS at baseline, median (IQR)	17 (10-20)	15 (11-19)	0.943
Hypertension, n (%)	53 (71.6)	333 (68.7)	0.608
Diabetes, n (%)	24 (32.4)	120 (24.7)	0.159
CHD, n (%)	5 (6.8)	61 (12.6)	0.148
Smoking, n (%)	30 (40.5)	179 (36.9)	0.547
Alcohol Drinking, n (%)	15 (20.3)	98 (20.2)	0.990
IV rTPA, n (%)	33 (44.6)	197 (40.6)	0.517
Lymphocyte (1000/mm3)	1.3 (1.0-1.8)	1.1 (0.8-1.6)	0.011
HbA1c (%)	6.4 (5.7-7.1)	5.9 (5.5-6.8)	0.036
HCY (mmol/l)	16.4(13.3-20.2)	14.6(12.1-18.7)	0.029
Pre-procedure ASPCET score	8 (7-9)	9 (8-9)	0.001
Pre-procedure ASITN/SIR score	1 (1-2)	3 (2-3)	<0.001
retriever passes >3 times, n (%)	12 (16.2)	40 (8.2)	0.028
TOAST, n (%)			
Large artery atherosclerosis	36 (48.6)	235 (48.5)	0.148
Cardioembolism	26 (35.1)	205 (42.3)
Other	12 (16.2)	45 (9.3)
Puncture-to-recanalization time, min, median (IQR)	80 (55-105)	67 (49-95)	0.014
mTICI score 2b or 3, n (%)	62 (83.8)	443 (91.3)	0.040
NIHSS 24 hours after procedure, median (IQR)	29 (17-35)	11 (5-17)	<0.001
sICH, n (%)	36 (48.6)	33 (6.8)	<0.001
MCE, n (%)	21 (28.4)	75 (15.5)	0.006
Favorable outcome, n (%)	8 (10.8)	276 (56.9)	<0.001
Mortality, n (%)	25 (33.8)	56 (11.5)	<0.001

NIHSS, National Institutes of Health Stroke Scale; CHD, coronary atherosclerotic heart disease; IV, intravenous thrombolysis; HbA1c, glycated hemoglobin; HCY, homocysteine; ASPECT, Alberta Stroke Program Early CT Score; ASITN/SIR, American Society of Interventional and Therapeutic Neuroradiology/Society of Interventional Radiology; TOAST, Trial of Org 10172 in Acute stroke treatment; mTICI, modified Thrombolysis in Cerebral Infarction; sICH, symptomatic intracranial hemorrhage; MCE, malignant cerebral edema.

**Figure 3:**
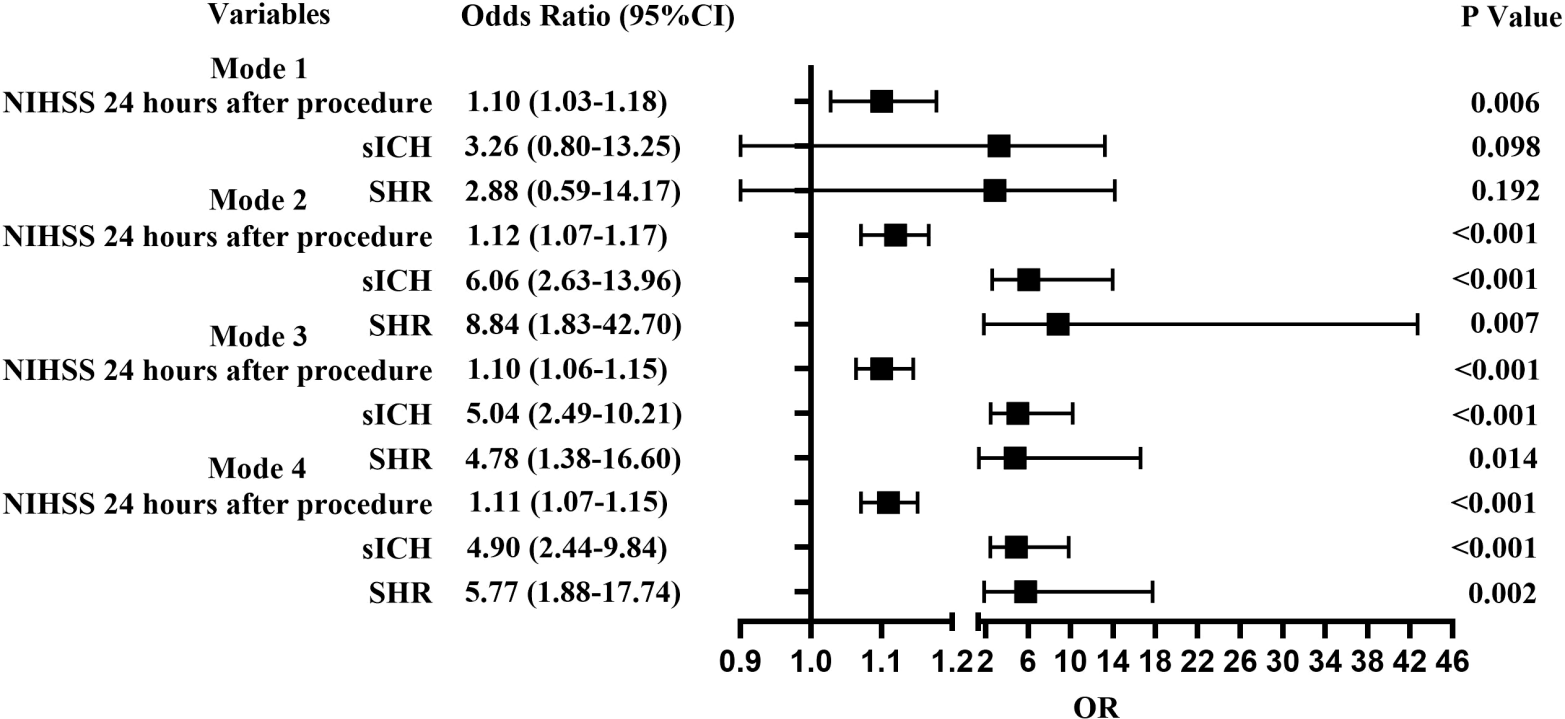
Forest plots for predictors of END. Model 1, Diabetic group; Model 2, Non-diabetic group; Model 3, Including fasting blood glucose and glycosylated hemoglobin as confounders; Model 4, Excluding fasting blood glucose and glycosylated hemoglobin as confounders.

In Model 3 with unadjusted fasting blood glucose and glycosylated hemoglobin being adjusted, SHR (OR =4.78; 95% CI, 1.38-16.60; P =0.014), sICH (OR =5.04; 95% CI, 2.49-10.21; P <0.001), and NIHSS score at 24 hours (OR =1.10; 95% CI, 1.06-1.15; P <0.001) were related to END. In Model 4 with fasting blood glucose and glycosylated hemoglobin being adjusted, SHR (OR =5.77; 95% CI, 1.88-17.74; P =0.002), sICH (OR =4.90; 95% CI, 2.44-9.84; P <0.001) and NIHSS score at 24 hours (OR =1.11; 95% CI, 1.07-1.15; P <0.001) were related to END (**Figure 3**).

When patients were stratified as with and without diabetes mellitus (DM) and adjusted for major confounding factors, multivariant analysis detected that sICH (OR =6.06; 95% CI, 2.63-13.96; *P <*0.001), 24-hour NIHSS score (OR =1.12; 95% CI, 1.07-1.17; *P <*0.001), and high SHR (OR =8.84; 95% CI, 1.83-42.70; *P* =0.007) could influence the development of END in patients without diabetes mellitus. High NIHSS score after 24 hours (OR =1.10; 95% CI, 1.03-1.18; *P* =0.006) could influence the development of END in patients with diabetes mellitus (**Figure 3**).

Multivariant analysis detected that SHR (OR =0.20, 95% CI, 0.08-0.50; *P* =0.001), age (OR =0.94, 95% CI, 0.91-0.98; *P* =0.001), baseline NIHSS score (OR =1.15, 95% CI, 1.05-1.25; *P* =0.002), NIHSS score after 24 hours (OR =0.76, 95% CI, 0.69-0.83; *P <*0.001), PRT (OR =0.99, 95% CI, 0.98-1.00; *P* =0.025), pre-procedure ASITN grade (OR =62.02, 95% CI, 25.92-148.38; *P <*0.001) and successful recanalization (OR =7.42, 95% CI, 1.94-28.31; *P* =0.001) were associated with favorable outcome (**Table 2**).

**Table 2:** Multivariable analysis for favorable outcome.

Variable	OR	95% CI	P-value
SHR	0.20	0.08-0.50	0.001
age	0.94	0.91-0.98	0.001
NIHSS at baseline	1.15	1.05-1.25	0.002
NIHSS 24 hours after procedure	0.76	0.69-0.83	<0.001
PRT	0.99	0.98-1.00	0.025
Pre-procedure ASITN/SIR	62.02	25.92-148.38	<0.001
mTICI, 2b-3	7.42	1.94-28.31	0.001

SHR, stress hyperglycemia ratio; NIHSS, National Institutes of Health Stroke Scale; PRT, puncture-to-recanalization time; ASITN/SIR, American Society of Interventional and Therapeutic Neuroradiology/Society of Interventional Radiology; mTICI, modified Thrombolysis in Cerebral Infarction.

## Discussion

This study observed that stroke patients with high SHR had increased incidence of END and decreased likelihood of favorable outcome after endovascular treatment.

SHR was determined as a better quantitative indicator for stress hyperglycemia than blood glucose level when evaluating the outcomes of critical illness (25). High SHR has been associated with increased risk of END and poor outcome in patients with intravenous thrombolysis. But no study on relationship between SHR and END has been reported in patients with endovascular thrombectomy (26). The underlying mechanism for SHR influencing END may be multifactorial. First, increased lactate productions may deteriorate ischemic condition, and disrupt neuron metabolism in penumbra areas (27). Second, stress hyperglycemia could aggravate hemorrhagic transformation after ischemic stroke by inducing mitochondrial dysfunction and endothelial cell apoptosis (28). Third, stress hyperglycemia may have adverse effects on collateral circulation (29). Fourth, the prothrombotic effect of stress hyperglycemia could result in thrombus extension and blood-brain barrier destruction (30).

Previous study confirmed that patients with high SHR had an increased risk of symptomatic intracranial hemorrhage and mortality after endovascular thrombectomy (15). This study associated SHR and unfavorable outcome in patients treated with endovascular thrombectomy. Several explanations may account for the association between SHR and unfavorable outcome after endovascular thrombectomy. First, acute stress response may lead to enhance inflammation reaction, which in turn leads to increased hepatic glycogenolysis, insulin resistance, cell endothelial injury, platelet aggregation, and mitochondrial dysfunction (11, 31). Second, stress hyperglycemia may directly damage ischemic brain tissue through lactic acid accumulation and intra-cellular acidosis, and aggravate ischemic injury (32). Third, stress hyperglycemia could generate reperfusion injury *via* oxidative stress and inflammatory process with increased expression of endothelial adhesion molecules and monomeric C-reactive protein (33). Fourth, stress hyperglycemia may disrupt blood-brain barrier and promote hemorrhagic transformation (34).

Several limitations of this study should be address when interpreting the results. END was defined as NIHSS score increase within 24 hours after endovascular procedures, but this condition could occur a few days later. We did not monitor dynamics changes of SHR. The effects of antidiabetic agents and anesthesia were not assessed.

High stress hyperglycemia ratio may be related to early neurological deterioration and decreased likelihood of favorable outcomes after endovascular thrombectomy in patients with acute ischemic stroke.

## Data availability statement

The datasets presented in this study can be found in online repositories. The names of the repository/repositories and accession number(s) can be found in the article/supplementary material.

## Ethics statement

The studies involving human participants were reviewed and approved by the ethics committees of the Affiliated Wuxi People’s Hospital of Nanjing Medical University and the Affiliated Nanjing Hospital of Nanjing Medical University. Written informed consent for participation was not required for this study in accordance with the national legislation and the institutional requirements.

## Author contributions

ZD and HC contributed equally to the conception of the research and drafted the manuscript. LL, HJ, and HG acquired the data. XZ, JZ, and FW analyzed the data. YJ revised the manuscript and made contribution to the revision, GX and DL revised the manuscript and approved the final version of the manuscript. All authors contributed to the article and approved the submitted version.
